# Simvastatin Combined with Antioxidant Attenuates the Cerebral Vascular Endothelial Inflammatory Response in a Rat Traumatic Brain Injury

**DOI:** 10.1155/2014/910260

**Published:** 2014-06-11

**Authors:** Kuo-Wei Wang, Hao-Kuang Wang, Han-Jung Chen, Po-Chou Liliang, Cheng-Loong Liang, Yu-Duan Tsai, Chung-Lung Cho, Kang Lu

**Affiliations:** ^1^Department of Neurosurgery, E-Da Hospital, I-Shou University, Kaohsiung City 824, Taiwan; ^2^Department of Biological Sciences, National Sun Yat-sen University, Taiwan

## Abstract

Traumatic brain injury (TBI) leads to important and deleterious neuroinflammation, as evidenced by indicators such as edema, cytokine production, induction of nitric oxide synthase, and leukocyte infiltration. After TBI, cerebral vascular endothelial cells play a crucial role in the pathogenesis of inflammation. In our previous study, we proved that simvastatin could attenuate cerebral vascular endothelial inflammatory response in a rat traumatic brain injury. This purpose of this study was to determine whether simvastatin combined with an antioxidant could produce the same effect or greater and to examine affected surrogate biomarkers for the neuroinflammation after traumatic brain injury in rat. In our study, cortical contusions were induced, and the effect of acute and continuous treatment of simvastatin and vitamin C on behavior and inflammation in adult rats following experimental TBI was evaluated. The results demonstrated that simvastatin combined with an antioxidant could provide neuroprotection and it may be attributed to a dampening of cerebral vascular endothelial inflammatory response.

## 1. Introduction

Traumatic brain injury (TBI) remains one of the leading causes of death and disability in industrialized countries. Despite numerous studies on animal models of TBI that have investigated therapeutic strategies, no effective therapy is currently available [1]. TBI leads to important and deleterious neuroinflammation, as evidenced by edema, cytokine production, induction of nitric oxide synthase, and leukocyte infiltration. Strategies that block inflammatory and oxidative mediators have been shown to induce neuroprotective and anti-inflammatory effects after brain injury [2].

After TBI, cerebral vascular endothelial cells play a crucial role in the pathogenesis of inflammation and it has been comprehensively reviewed [3]. In this study, we chose to assay ICAM-1 and IL-10 as the markers of vascular endothelial cell inflammation according to the result of our previous study [3]. Statins, a class of lipid-lowering drugs, inhibit 3-hydroxy-3-methylglutaryl-CoA reductase, thereby suppressing cholesterol biosynthesis. Apart from their lipid-lowering activities, statins have been shown to mediate pleiotropic effects* in vitro* and* in vivo* by reducing inflammation and oxidative stress [4, 5]. Several studies have shown that the administration of statins induced neuroprotective and anti-inflammatory effects and improved neurological outcomes after experimental TBI [3, 6–9].

Vitamin C in human must be ingested for survival. It is an electron donor, and the property accounts for all its known functions. The antioxidant effects of vitamin C have been demonstrated in many experiments* in vitro*. Human diseases such as atherosclerosis and cancer might occur in part from oxidant damage to tissues. The relationship of this oxidant to human disease conditions is not very clear at this time, but it has been studied [10–12].

To date few published studies have investigated the consequences of a combination therapy following TBI, and that it may decrease the side effects of the single drug. In this study, we investigated the effect of acute and continuous treatment of simvastatin combining with vitamin C on behavior and inflammation in adult rats following experimental TBI.

## 2. Materials and Methods

### 2.1. Animals

All experiments were approved by the Institutional Animal Care and Use Committee (IACUC) of E-DA Hospital and complied with the IACUC Guide for the Care and Use of Laboratory Animals. Adult male Sprague-Dawley rats (*n* = 30; weight: 400–450 g) were group-housed on a 12-12 h light-dark cycle and were provided with standard diet.

### 2.2. TBI in Rats

Cortical contusions were induced using a device adapted from the impact method described in detail elsewhere [13, 14]. Briefly, rats were anesthetized with halothane, body temperature was maintained at 37°C, and other vital signs were held stable. The scalp was cleaned with Ioprep, and aseptic techniques were used throughout surgery. The scalp was opened, and a craniotomy was performed over the left hemisphere; the center of the footplate was positioned 1.5 mm posterior and 2.5 mm lateral to the bregma [14, 15]. Contusions were made in the “hind paw” area, which consists of overlapping motor and somatosensory fields [16]. This area was selected because it is readily accessible and relatively flat, and if injured, it produces a readily observable deficit. Animals were randomly assigned as unilateral contusion or craniotomy controls.

Following the removal of a small bone flap, a stainless steel circular footplate was placed so that it rested just upon the surface of dura, which remained intact. To prevent contused cortex from herniating into the opening, craniotomies were only slightly larger than the diameter of the footplate. A 40 cm long stainless steel tube kept at a 90° angle was used to guide a falling 20 g brass weight onto the footplate. To prevent air compression in the tube, the tube was perforated at 1 cm intervals.

Following surgery, animals were placed in the prone position on a 10 cm foam block, and the foam block was placed beneath the contusing device. Injury was induced by release of a 20 g brass weight from a height of 40 cm onto the foot plate. The degree of injury was created by repeated controlled cortical impacts, and the injured rats received 10 cortical impacts. After impact, the bone flap was replaced and sealed with bone wax, the scalp sutured closed, and the animals were allowed to recover.

### 2.3. Experimental Protocol

There were 5 groups utilized for the study: (1) sham group, craniotomy only (*n* = 6); (2) control group, TBI without treatment (*n* = 6); (3) treatment group (*n* = 6), TBI with vitamin C, administration only; (4) treatment group (*n* = 6), TBI with simvastatin only; and (5) treatment group (*n* = 6), TBI with combination therapy. According to our previous study and other studies (Shao et al. [17]), we chose to administer an even higher dose (15 mg/kg) of simvastatin (Merck). The treatment group received 15 mg/kg of simvastatin in 1 mL of distilled water daily and vitamin C (20 mg/kg) (Chi Sheng) for 3 days via an orogastric tube inserted each day [18]. The first dose was given 1 h after experimental TBI. Each animal in the control and sham groups received 1 mL/day of distilled water via the same route [19]. The rats were sacrificed 7 days later and brain specimens were preserved for immunochemistry analysis.

### 2.4. Neurological Score

Neurological function was assessed with the grip test (grip strength meter, Singa). The grip test was performed before TBI induction and then at 24 h, 72 h, and 1 week after TBI. We evaluated the muscle power of all 4 limbs, and a trial was successful if the effective grip power of limbs was more than 3 g. We recorded the frequency of successful trials in 60 seconds [20].

### 2.5. Determination of ICAM-1 and IL-10 Levels

Blood samples were collected in tubes with potassium acetate before injury and at selected times after injury (24, 48, and 72 h and 7 days). Samples were then centrifuged at 3000 ×g for 5 min, immediately frozen, and stored at −80°C. The ICAM-1 level was measured using commercially available quantitative sandwich enzyme-linked immunosorbent assay (ELISA) kits (R&D System, USA).

### 2.6. Statistical Analysis

Data shown in figures are presented as mean ± SEM. Repeated measure ANOVA with robust standard error and exchangeable working correlation matrix (compound symmetry) of generalized estimating equation (GEE) was used to determine the *P* values for the main effects of time and treatment and interaction effects of time by treatment. When a significant interaction effect of time by treatment was revealed, simple main effects (pairwise comparisons) were calculated with GEE within-model contrast (LSD method). In the analysis of simple main effects, comparisons among groups at a specific time point were performed. SPSS software package 15.0 for Windows was used for all analyses. *P* < 0.05 was considered to be statistically significant.

## 3. Results

**Figure 1:**
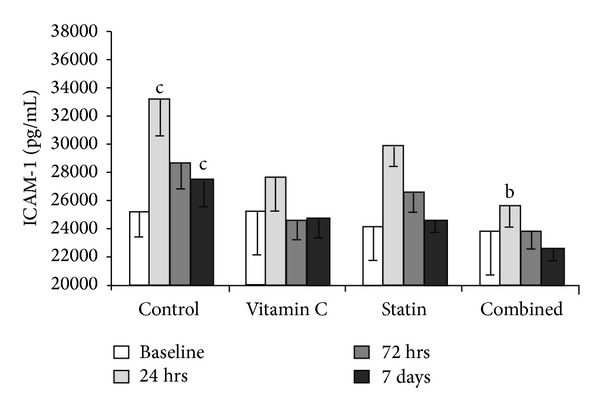
The labels “a,” “b,” and “c” denote *P* ≤ 0.05 for the comparison versus vitamin C group, statin group, and combined group at a specific time-point, respectively. In the time- point of 24 h and day 7 the combination group showed a significant reduction in ICAM-1 as compared to the control group. In the time-point of 24 h the combination group had a significant reduction in ICAM-1 when compared to the statin group. However, there was no difference in each time-point between vitamin C and combination group.

In Figure 1, we present the results of biomarker findings of inflammation after traumatic brain injury and the treatment groups had a significant reduction in ICAM-1. There was no result in IL-10. In the time-point of 24 h and day 7 the combination group showed a significant reduction in ICAM-1 as compared to the control group. In the time-point of 24 h the combination group had a significant reduction in ICAM-1 when compared to the statin group. However, there was no difference in each time-point between vitamin C and combination group.

**Figure 2:**
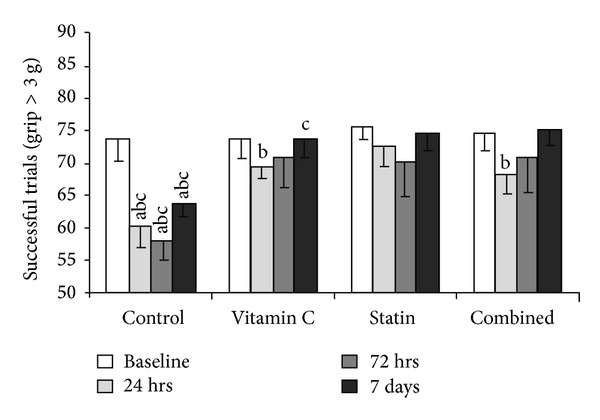
The labels “a,” “b,” and “c” denote *P* < 0.05 for the comparison versus vitamin C group, statin group, and combined group at a specific time-point, respectively. In the time- point of 24 h the statin group had the best neurological function when compared to the other 3 groups. At the time-point of the 7th day the combination group had better performance in neurological function when compared to the vitamin C group.

In Figure 2, we present the fact that all treatment groups had better grip test than the control group for 24 h, 72 h, and the 7th day, respectively. In the time-point of 24 h the statin group had the best neurological function when compared to the other 3 groups. At the time- point of the 7th day the combination group had better performance in neurological function when compared to the vitamin C group. In contrast, there was no difference in each time-point between the combination group and statin group.

**Figure 3:**
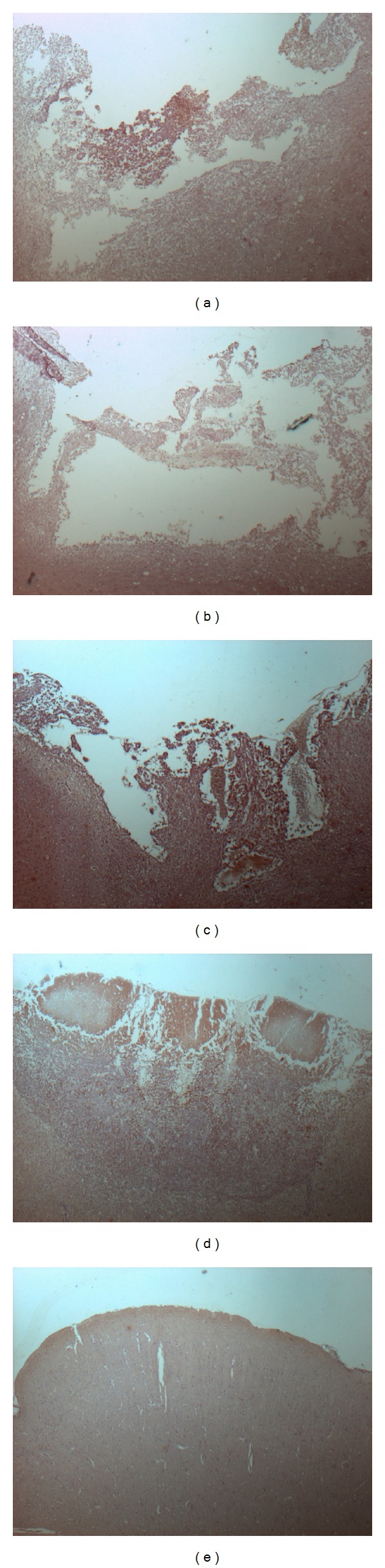
(a) The control group showed severe cortex injury and brain tissue loss. (b) The simvastatin treatment group showed some cortex injury and brain tissue loss. (c) The vitamin C treatment group also showed some cortex injury and brain tissue loss. But the area of injury was smaller. (d) The combination treatment group showed mild cortex injury and minimal brain tissue loss. (e) The sham group showed intact cortex.

In Figures 3(a), 3(b), 3(c), 3(d), and 3(e), the result of immunochemistry staining showed mild injury and minimal brain tissue loss in combination treatment group when compared with control group and other treatment groups. Smaller area of loss of brain tissue was noted in vitamin C group when compared with simvastatin treatment group.

## 4. Discussion

In this study, we have shown that treatment with vitamin C, simvastatin, or combination therapy could attenuate the cerebral vascular endothelial inflammatory response in a rat traumatic brain injury and reduce neurological deficit after traumatic brain injury. At the time- point of 24 h the combination group showed a significant reduction in ICAM-1 when compared to the statin group. But there was no difference between the vitamin C and combination group. All treatment groups had better grip test than the control group at 24 h, 72 h, and on the 7th day, respectively. Our findings suggested that vitamin C and combination therapy could play a critical role in the TBI-mediated inflammatory response and reduce the neurological deficit.

The clinical and experimental studies of antioxidant and anti-inflammatory effects of vitamin C have been reported and the antioxidant effects of vitamin C on vascular endothelium have been proposed but not studied in traumatic brain injury. Vitamin C may increase endothelial nitric oxide (NO) by protecting it from oxidation and increasing its synthesis [21, 22]. Vitamin C and the other antioxidant vitamin, vitamin E, appear to have beneficial effects on vascular endothelial function in healthy subjects and in patients with cardiovascular disease [23, 24]. In healthy subjects, vitamin C administration restored endothelium-dependent vasodilation that was impaired by acute hyperglycemia [25]. Thus vitamin C may have favorable effects on vascular dilatation, possibly through its antioxidant effects on NO, but these findings are not consistent [26–28]. In stroke study vitamin C may play a critical role in stroke-mediated inflammatory response and may be associated with neurological changes and cognitive impairment [12]. In different animal models antioxidant depletion was observed after focal cerebral ischemic brain injury. A number of factors may be responsible for this phenomenon, including the physiological need of glial cells, particularly astrocytes, coupled with the removal of increased levels of glutamate after stroke. Another factor may be the formation of free radicals after stroke. In our study we proposed that vitamin C could attenuate the vascular endothelial inflammatory response after traumatic brain injury in rats with the reduction of ICAM-1 level and it was never mentioned before.

ICAM-1 plays a critical role in mediating cell-cell contact between leukocytes and cells of various origins. ICAM-1 expression is upregulated by inflammatory cytokines and appears to play a critical role in the posttrauma inflammatory response [2]. Endothelial activation plays an important role in the pathophysiology of the inflammatory reaction and vascular injury after traumatic injury. After injury ICAM-1 interacts with leukocyte integrins to mediate firm adhesion of activated neutrophils to endothelium and diapedesis [4–7]. ICAM-1 also contributes to brain leukocyte accumulation and leukocyte-mediated tissue injury in experimental models of stroke, meningitis, and systemic trauma [4–7].

IL-10 is a member of the interferon/IL-10 family and it is designated as an anti-inflammatory cytokine. It inhibits proinflammatory cytokine production and stimulates cytotoxic T-cell development and B-cell proliferation [24]; we chose to use ICAM-1 and IL-10 as markers of endothelial activation and inflammation.

Our results demonstrated that all treatment groups could attenuate the expression of ICAM-1, but not IL-10. At these time-points IL-10 expression declined to basal or below basal levels after its maximum increase and after traumatic brain injury [2, 12]. The effect of simvastatin for TBI has been confirmed, but the effects of vitamin C and such combination therapy have not been discussed. Our data showed that these two groups could attenuate the vascular endothelial inflammatory response, but there was no difference between these two groups in some time- points. It may be the effect of dose. We chose a higher dose of simvastatin and a lower dose of vitamin C [12, 18]. From previous studies with vitamin C, a higher dose of vitamin C was used and it could be in the range of 50 mg/100 g [29–31]. As a logical next step we would like to adjust the dose of vitamin C and simvastatin and investigate the effect of vitamin C at a higher dose.

### 4.1. Limitation

There are some limitations about the study. First this is an experimental study and we can control the timing of treatment. But in clinical condition the acute treatment about traumatic brain injury is started several hours later even more. Second, we treated higher dose of simvastatin in traumatic brain injury of rat. But we cannot prescribe such higher dose to human patients. However, we confirmed that such treatment could reduce neuroinflammation after traumatic brain injury.

## 5. Conclusion

This is the first study showing the efficacy of a simvastatin-vitamin C combinational therapeutic approach in achieving molecular, histological, and neurological recovery after TBI. Our results showed that such combinational therapy could attenuate the cerebral vascular endothelial inflammatory response in a rat traumatic brain injury. Another finding was that vitamin C also could attenuate the vascular endothelial inflammatory response.
